# The effects of the Er:YAG laser on trabecular bone micro-architecture: Comparison with conventional dental drilling by micro-computed tomographic and histological techniques

**DOI:** 10.12688/f1000research.12018.1

**Published:** 2017-07-17

**Authors:** Jihad Zeitouni, Bret Clough, Suzanne Zeitouni, Mohammed Saleem, Kenan Al Aisami, Carl Gregory

**Affiliations:** 1 American Board of Laser Surgery, Trumbull, CT, USA; 2 Department of Medical Physiology, College of Medicine, Texas A&M Health Science Center, Temple, TX, USA; 3 Department of Molecular and Cellular Medicine, Institute for Regenerative Medicine, College of Medicine, Texas A&M Health Science Center, College Station, TX, USA; 4 Dental Royal Complex, Dammam, Saudi Arabia

**Keywords:** dental drilling, Er:YAG laser, micro-computed tomography

## Abstract

**Background**: The use of lasers has become increasingly common in the field of medicine and dentistry, and there is a growing need for a deeper understanding of the procedure and its effects on tissue. The aim of this study was to compare the erbium-doped yttrium aluminium garnet (Er:YAG) laser and conventional drilling techniques, by observing the effects on trabecular bone microarchitecture and the extent of thermal and mechanical damage.

**Methods**: Ovine femoral heads were employed to mimic maxillofacial trabecular bone, and cylindrical osteotomies were generated to mimic implant bed preparation. Various laser parameters were tested, as well as a conventional dental drilling technique. The specimens were then subjected to micro-computed tomographic (μCT) histomorphometic analysis and histology.

**Results**: Herein, we demonstrate that mCT measurements of trabecular porosity provide quantitative evidence that laser-mediated cutting preserves the trabecular architecture and reduces thermal and mechanical damage at the margins of the cut. We confirmed these observations with histological studies. In contrast with laser-mediated cutting, conventional drilling resulted in trabecular collapse, reduction of porosity at the margin of the cut and histological signs of thermal damage.

**Conclusions**: This study has demonstrated, for the first time, that mCT and quantification of porosity at the margin of the cut provides a quantitative insight into damage caused by bone cutting techniques. We further show that with laser-mediated cutting, the marrow remains exposed to the margins of the cut, facilitating cellular infiltration and likely accelerating healing. However, with drilling, trabecular collapse and thermal damage is likely to delay healing by restricting the passage of cells to the site of injury and causing localized cell death.

## Introduction

Since the pioneering work of Stern, Sognnaes and the Goldman brothers on the ruby laser in the 1960s, followed by the CO _2_ and Nd:YAG lasers in the 1980s ( Coluzzi & Convissar, 2004; Featherstone & Nelson, 1987), and the erbium series of lasers in 1989 ( Hibst & Keller, 1989), there has been considerable interest in the use of laser radiation for cutting of bone tissue, particularly in the field of dentistry.

Over the past ten years, the Er:YAG laser with a working wavelength of 2940 nm is one of the most commonly used in dentistry ( Romanos, 2015). It has been suggested that the Er:YAG laser is probably the least destructive of the bone cutting lasers because it generates light at an energy level that is readily absorbed by water and thus minimizes carbonation and adjacent tissue necrosis ( Bornstein, 2003). While a handful of studies have suggested that Er:YAG laser energy is indeed sparing of tissue ( Baek *et al.*, 2015; Gholami *et al.*, 2013; Panduric *et al.*, 2014; Yoshino *et al.*, 2009), the field is controversial, with at least one study predicting that laser energy causes excessive thermal damage ( Martins *et al.*, 2011). Furthermore, studies that specifically address the microstructure of bone after exposure to laser radiation are qualitative.

To help address these concerns, we propose a method to quantitatively evaluate thermal and mechanical destruction of trabecular bone by cutting techniques, and ask definitively whether the Er:YAG laser causes less thermal tissue damage than conventional drilling techniques. Motivated by forensic studies ( Thompson, 2005), we reasoned that trabecular structure would collapse during thermal or mechanical challenge, and this could be quantified by standard measures of porosity. Moreover, this assay could be rapidly performed with standard modern μCT and histomorphometry methodologies. Herein, we compare the effect of typical Er:YAG laser parameters with conventional drilling techniques on trabecular microarchitecture with μCT scanning, computational histomorphometry and histology.

## Methods

### Experimental specimens

Femoral heads of 1 year-old lambs were acquired from a meat distributer (Antonis Butchers, Paralimni, Cyprus) and used within five days of acquisition. Ethical approval was not required in this case because the specimens utilized were from pre-existing biological material, rather than from animals euthanized for the purpose of a scientific study. The articular surface of the femoral head was thoroughly cleaned and then covered by a 3 mm layer of silicone to prevent contamination by outside particulates. Guide holes were made in the silicone that ensured that the diameter of the hole created by the laser was consistent across all samples. Using the various means described below, cylindrical osteotomies were created 4 mm diameter by 5 mm depth to mimic a typical implant bed. The laser (Lambda Pluser, Brendola, Italy) was used to create the three osteotomies using three typically utilized settings, designated hereafter as condition 1, 2 and 3 ( Table 1– Table 3). To compare to a conventional drilling technique (Bicon Drill System, Bicon, Boston, MA, USA), an osteotomy was generated with a 2 mm pilot drill at 1250 rpm with irrigation, followed by enlargement at 50 rpm in the absence of irrigation. As a positive control, to validate porosity measurements and histological observations, an abrasive diamond-coated dental burr (Strauss, model 836KR, Palm Coast, FL, USA) was also used, which provided highly damaged reference material for comparison with experimental samples. Negative control blocks that did not receive holes were also prepared. Blocks of bone (10×10×10 mm) harboring each hole were cut from the femoral head with a diamond coated rotary blade (0.2 mm by 15 mm diameter, Strauss Diamond) fitted to a heavy duty drill (Foredom K5300 Blackstone Industries, Bethel, CT, USA).

**Table 1.** Laser parameters for condition 1.

**Intrinsic Parameters**		**Adjustable Parameters**		**Calculated Parameters**	
**Manufacturer**	Lambda	**Average Power** **(watts)**	2.5	**Energy per pulse** **(mj)**	250
**Model**	Pluser	**Energy per pulse** **(mj)**	250	**Average Power** **(watts)**	2.5
**Type**		**Pulse width** **(microsec)**	75	**Peak Power** **(watts)**	3.333 watts
**Wavelength (nm)**	2940	**Pulse repetition** **rate (PPS)**	10	**Tip Area (cm ^2^)**	0.0050
**Delivery System (Fiber,** **sapphire tip, articulated** **arm)**	Sapphire tip	**Tip diameter (um)**	800	**Spot Diameter at** **Tissue (cm)**	0.1362
**Emission Mode** **(continuous wave, gated,** **free running pulse)**	Free running pulse	**Tip-to-Tissue** **(millimeters)**	2	**Spot Area at** **Tissue (cm ^2^)**	0.0146
**Energy Distribution** **(Gaussian or flat-top)**	Gaussian	**Beam divergence** **(degrees)**	8	**Peak Power** **Density (w/cm ^2^)**	228,734
**Tip initiation**	none	**Water (ml/min)**	24	**Average Power** **Density (w/cm ^2^)**	172
**Initiation technique**	none	**Air (ml/min)**	none	**Pulse Energy** **Density (j/cm ^2^)**	367
		**Length of** **treatment (sec)**	2100	**Total Energy** **(joules)**	5250

**Table 2.** Laser parameters for condition 2.

Intrinsic Parameters		Adjustable Parameters		Calculated Parameters	
**Manufacturer**	Lambda	**Average Power** **(watts)**	8	**Energy per pulse** **(mj)**	400
**Model**	Pluser	**Energy per** **pulse (mj)**	400	**Average Power** **(watts)**	8
**Type**		**Pulse width** **(microsec)**	75	**Peak Power** **(watts)**	5.333
**Wavelength (nm)**	2940	**Pulse repetition** **rate (PPS)**	20	**Tip Area** **(cm ^2^)**	0.0050
**Delivery System** **(Fiber, sapphire tip,** **articulated arm)**	Sapphire tip	**Tip diameter (um)**	800	**Spot Diameter at** **Tissue (cm)**	0.1362
**Emission Mode** **(continuous wave,** **gated, free running** **pulse)**	Free running pulse	**Tip-to-Tissue** **(millimeters)**	2	**Spot Area at Tissue** **(cm ^2^)**	0.0146
**Energy Distribution** **(Gaussian or flat-top)**	Gaussian	**Beam divergence** **(degrees)**	8	**Peak Power Density** **(w/cm ^2^)**	365.975
**Tip initiation**	none	**Water (ml/min)**	24	**Average Power** **Density (w/cm ^2^)**	549
**Initiation technique**	none	**Air (ml/min)**	none	**Pulse Energy** **Density (j/cm ^2^)**	1175
		**Length of** **treatment (sec)**	3600	**Total Energy** **(joules)**	2880

**Table 3.** Laser parameters for condition 3.

**Intrinsic Parameters**		**Adjustable Parameters**		**Calculated Parameters**	
**Manufacturer**	Lambda	**Average Power** **(watts)**	6	**Energy per pulse** **(mj)**	400
**Model**	Pluser	**Energy per pulse** **(mj)**	400	**Average Power** **(watts)**	6
**Type**		**Pulse width** **(microsec)**	75	**Peak Power (watts)**	5.333
**Wavelength (nm)**	2940	**Pulse repetition** **rate (PPS)**	15	**Tip Area (cm ^2^)**	0.0050
**Delivery System** **(Fiber, sapphire tip,** **articulated arm)**	Sapphire tip	**Tip diameter** **(um)**	800	**Spot Diameter at** **Tissue (cm)**	0.1362
**Emission Mode** **(continuous wave,** **gated, free running** **pulse)**	Free running pulse	**Tip-to-Tissue** **(millimeters)**	2	**Spot Area at** **Tissue (cm ^2^)**	0.0146
**Energy Distribution** **(Gaussian or flat-top)**	Gaussian	**Beam divergence** **(degrees)**	8	**Peak Power Density** **(w/cm ^2^)**	365,975
**Tip initiation**	none	**Water (ml/min)**	24	**Average Power** **Density (w/cm ^2^)**	412
**Initiation technique**	none	**Air (ml/min)**	none	**Pulse Energy** **Density (j/cm ^2^)**	881
		**Length of** **treatment (sec)**	240	**Total Energy (joules)**	1440

### Micro-computed tomography (µCT)

With the holes in the vertical orientation, the bone blocks were scanned at 40 kV/661 mA at 21 mm resolution using a Skyscan 1174 µCT unit (Bruker, Kontich, Belgium). Data were collected at 1° increments over the 360° with flat field, random movement and geometrical correction activated. After acquisition, the data were thresholded to a scale ranging between 350 and 2554 Hounsfield units, so as to maximize visualization of trabecular bone. Axial images corresponding to 20 μm sections were then obtained using NRecon software (Vers 1.5.1.1, Skyscan) and saved as JPEG files.

**Figure 1.**
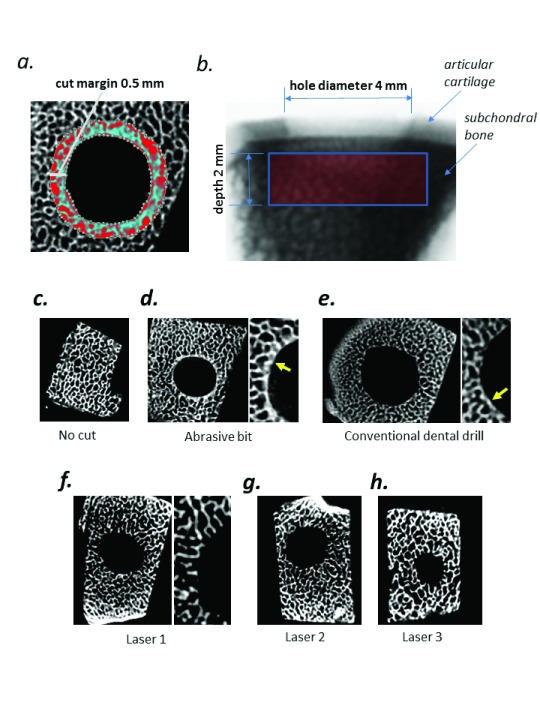
**a**) The ROI plotted on each of the 10 axial sections encompasses a 0.4 – 0.5 mm margin around the circumference of each hole. **b**) Measurements are taken on every 10 ^th^ section from the surface of the hole to a depth of 2 mm, resulting in 10 values. **c**) Negative (uncut) control demonstrating distinct trabecular architecture. **d**) Left panel: demonstrating a dense layer along the circumference of the hole caused by thermal damage due to friction (arrowed). Right panel: magnified image illustrating a dense compacted layer (arrowed). **e**) Left panel: hole cut with conventional dental drill, with similar dense layer. Right panel: magnified image illustrating a compacted layer (arrowed). **f**) Left panel: hole cut with laser at 2.5 Watts, demonstrating undamaged trabecular structures at the circumference of the hole. Right panel: magnified image. **g**) Laser condition 2 (6 Watts). **h**) Laser condition 3, (8 Watts).

In an attempt to objectively quantify damage to bone, the change in trabecular porosity was measured at the margin of the cut. Trabecular structures collapse under extreme heat and abrasion caused by conventional drilling ( Thompson, 2005) ( Heinemann *et al.*, 2012). This results in a reduction in the porosity of trabecular bone which can be employed to quantify thermal and mechanical damage. To perform these measurements, a region of interest (ROI) was plotted on axial sections corresponding to a 0.4 – 0.5 mm margin around the hole ( Figure 1a). This ROI was plotted on every 10 ^th^ section from the surface of the hole to a point 2 mm below the surface ( Figure 1b). The percent porosity was calculated on 10 × 20 μm sections along a 0.4 – 0.5 mm thick margin at the edge of each hole ( Figures 1a, b) using CTAn software (Vers 1.8.1.4, Skyscan), and the means and standard deviations were calculated using GraphPad Prism version 5.00 for Windows (GraphPad Software, California, USA). Multiple pairwise comparisons within datasets were analyzed using one-sided ANOVA followed by Dunnet’s post-test. P-values below 0.05 were designated statistically significant in all cases. Statistical tests and data plotting were performed using GraphPad.

### Histology

Following μCT measurements, bone blocks were washed with fresh saline and decalcified in 1M dibasic ethylene-diamine tetra-acetic acid at pH 8.0 for 4 weeks, then with 8% (v/v) formic acid for a further 5 days (Sigma, St Louis, MO, USA) until radiolucency. The tissue was chemically dehydrated through an ascending gradient of alcohols and was then cleared with Sub-X clearing agent (Surgipath Medical Industries Inc., Richmond, IL). Paraffin-embedded blocks (paraffin wax type 6, Richard-Allan Scientific; Kalamazoo, MI) were cut in 10 μm thick sections and floated onto Superfrost plus microscope slides (Fisher Scientific). Sections were baked at 60°C for one hour before clearing with citrus clearing agent (Richard-Allan Scientific) and rehydration with distilled water. Masson’s trichrome staining was performed using a commercially acquired kit (American Mastertech Scientific Inc., Lodi, CA). Permount with toluene (Fisher Scientific was used as a mounting medium. Micrographs were generated using an upright microscope (Nikon Eclipse 80i fitted with a Retiga 2000 camera) running digital imaging software (Elements Vers 4.20, Nikon).

## Results

MicroCT scanning of bone blocks revealed a classical trabecular bone structure that was readily visualized in axial reconstructions ( Figures 1c–h). Upon inspection of the margin of the hole drilled with the abrasive bit (positive control) a distinct layer of compacted trabecular bone was evident, suggestive of heat damage ( Figure 1d). This layer was evident albeit to a lesser extent along the edges of the hole generated by the conventional dental drill ( Figure 1e). Conversely, the trabecular structures were preserved along the edges of the holes, generated by all 3 laser conditions ( Figures 1f–h).

**Figure 2.**
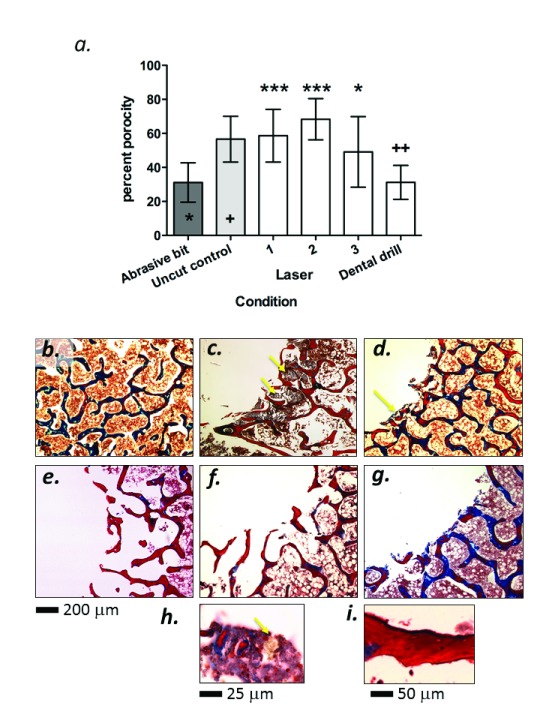
**a**) Plot of porosity measurements with statistical analyses. Values represent mean porosity for the 10 measured sections per sample with error bars representing standard deviations (n=10). Statistics are one-sided ANOVA with Dunnett’s post-test. Asterisks refer to comparison with abrasive bit (p<0.005=***, p<0.05=*). Crosses refer to comparison with negative (uncut) control (p<0.01=++). Panels **b**– **g** represent trichrome stained 10 mm sections of cut margins. **b**) Uncut control bone. **c**– **d**) Bone cut with abrasive bit and dental drill respectively, demonstrating areas of destroyed trabecular bone with severe carbonization (arrowed). **e**– **g**) Bone cut with laser parameters 1–3 respectively, demonstrating a lack of trabecular compaction and clean margins. **h**) 100× original magnification of charred cell mass (arrowed) present extensively in dental drill and abrasive bit. **i**) 60× original magnification of sporadic areas of slight carbonization that occurs with the laser.

To quantify the extent of the damage caused by cutting, the percentage porosity was measured on 10 × 20 μm sections along a 0.4 – 0.5 mm thick margin at the edge of each hole ( Figures 1a, b). The percentage porosity is reduced in compacted trabecular bone, providing a surrogate measure of heat and abrasive damage. Under the conditions of measurement described in the methods section, the negative control (uncut) bone sample ROI had a mean porosity of 56% ( Figure 2a). In contrast, the abrasive diamond bit (positive control) sample ROI had approximately half the porosity seen in the uncut control ( Figure 2a). When the experimental samples were measured, it was apparent that the hole generated by the conventional dental drill had a porosity significantly lower than the uncut control (p<0.01 indicated by ++ on the histogram in Figure 2a), but statistically indistinct from the specimen cut with the abrasive test bit. In contrast, those holes generated by laser had a trabecular porosity that was statistically similar to uncut bone, with condition 1 and 2 exhibiting the highest porosities (p<0.005 compared to positive control, indicated by *** on the histogram in Figure 2a) and condition 3 showing slightly lower porosity but still statistically distinct from the abrasive control (p<0.05, indicated by * on the histogram in Figure 2a). Collectively, these data demonstrate that the laser preserves the trabecular structure at the margin of cuts, whereas conventional drilling causes trabecular compaction, probably due to thermal or abrasive damage.

The bone samples were then decalcified, paraffin embedded and subjected to histological analysis. Uncut bone ( Figure 2b) had a distinct trabecular appearance when stained with Masson’s Trichrome, demonstrating areas of mature (blue) and remodeling (red) osteoid, typical of homeostatic bone tissue. Conversely, holes cut with the abrasive bit indicated distinct signs of trabecular collapse at the margin of the hole, with clear signs of severe carbonization on the bone tissue ( Figure 2c, arrowed) and also in the marrow cavities adjacent to the cutting site. Localized carbonization was also detected on the sample cut with the conventional dental drill, but to a lesser degree than the abrasive bit ( Figure 2d, arrowed). When visualized at high power, clusters of carbonized cells and charred debris were evident ( Figure 2h). All holes cut with the laser lacked significant signs of carbonization and where evident, it was minor and sporadic ( Figures 2f–g). Qualitatively, the carbonization appeared to increase with increasing laser power ( Figure 2h), but even at the highest setting, the carbonization was not as severe as the abrasive bit or the conventional dental drill.

## Discussion

Laser technology is potentially an attractive alternative to mechanical and electrosurgical approaches for dental osteotomy, but there is a lack of comparative preclinical and clinical studies ( Ishikawa *et al.*, 2008; Moslemi *et al.*, 2017). Nevertheless, it has been suggested that the Er:YAG laser is particularly suited to dental applications because the wavelength of the light employed has the capacity to cut hydroxyapatite, but the energy is readily absorbed by water, thus minimizing fear of soft tissue damage ( Bornstein, 2003). A recent study compared the Er:YAG laser to standard mechanical cutting techniques on porcine rib explants, and demonstrated that the laser generated a cut with well-defined trabecular spaces at the margin. In contrast, drilling resulted in what was described by the investigators as a “smear-like surface” with no clear trabecular patterning ( Panduric *et al.*, 2014). The investigators also reported virtually no carbonization at energies in excess of those employed in this study (1000 mJ versus 250–400 mJ). Later, Baek *et al.* reported the same qualitative differences in bone micro-architecture at the cut margin, when targeting the mandibular ridge of live porcine subjects ( Baek *et al.*, 2015). The Baek study further proposed that the open architecture of the cut margin could facilitate bleeding which in turn could facilitate healing. While highly informative, the Panduric and Baek studies employed electron microscopy to evaluate the cut margin and data were limited to quantitative evaluation. The results presented here corroborate the findings of both reports, but we also offer the novel contribution of a quantitative appraisal of bone architecture.

We reasoned that extreme exposure to thermal and abrasive energy would result in local trabecular collapse that could be measurable as a function of reduced porosity. Indeed, the compaction of trabecular structure is a well-known forensic indicator of bones subjected to excessive heat ( Thompson, 2005). Using high resolution μCT scans, it was possible to define an ROI ( Figure 1a, b) that corresponded to the cut margin in cylindrical osteotomies performed with 3 standard laser parameters, a conventional dental drill and a highly abrasive diamond bit. We then employed standard histomorphometric software to measure the porosity in 10 virtual axial cross sections for each condition. In support of the rationale, we found that the highly abrasive diamond bit caused significantly reduced trabecular porosity as compared to uncut bone ( Figure 2a). Furthermore we found that conventional drilling caused more trabecular compaction than all of the laser conditions ( Figure 2a). There were no statistically significant differences in trabecular porosity between laser energies employed.

Another sign of heat damage is carbonization. Examination of the histological sections showed localized carbonization presence on the sample cut with the conventional dental drill ( Figure 2c, h) and extensively carbonized tissue with the abrasive bit ( Figure 2d). All experimental samples cut with the laser lacked significant signs of carbonization ( Figures 2e–g), but at high laser energy, a thin carbonized layer was evident on some surfaces ( Figure 2i).

While the data presented here and the work of the aforementioned groups suggest that the Er:YAG laser results in minimized deformation of bone tissue and accelerated healing, a contrasting study suggests that Er:YAG cuts could slow healing through thermal damage of a thin layer of tissue ( Martins *et al.*, 2011). While surprising, the reason for these contrasting observations probably arises from distinctions between the structure of the bone tissues analyzed. In the Martins study, a qualitative appraisal was made on cortical bone of rodents, whereas the Baek study and the data presented here, focus on the structure of trabecular bone, which is more typical of the structure of the mandible in larger animals, including humans. We suggest that cortical bone offers a flat, uninterrupted surface for accumulation of thermal damage whereas the complex surface of trabecular bone would be expected to mask a significant area from the direct effects of the electromagnetic radiation.

The evidence presented in this study suggests that the use of the Er:YAG laser preserves trabecular architecture at the cut margin and is therefore likely more suitable for osteotomy than the conventional dental hand-piece. We also propose that a combination of μCT scanning and measurement of cut margin porosity represents a useful quantitative measure of thermal and mechanical destruction caused by bone-cutting tools. Further studies are needed to confirm these predictions in live animal subjects.

## Data availability

**Available raw datasets on Open Science Framework,** DOI, 10.17605/OSF.IO/PB8V9 ( Gregory, 2017):

- **‘Abrasive drill’:** Raw scans of the bone blocks, cut with the abrasive tool
- **‘Uncut’: Raw scans of uncut bone**
- **Dental drill:** Raw scans of the bone blocks, cut with the dental drill
- **Laser condition 1:** Er:YAG laser condition 1
- **Laser condition 2:** Er:YAG laser condition 2
- **Laser condition 3:** Er:YAG laser condition 3
- **Uncropped images of Figure 2h and Figure 2i.**

## Supplementary material

Supplementary File 1: Percent porosity calculated from the raw data used to generate Figure 2a.

Click here to access the data.
