# Supplementary material for: The effects of the Er:YAG laser on trabecular bone micro-architecture: Comparison with conventional dental drilling by micro-computed tomographic and histological techniques
Source: F1000Res. 2017 Jul 17;6:1133. [Version 1] doi: 10.12688/f1000research.12018.1 (PMC5782408; doi:10.12688/f1000research.12018.1)
Supplement: Peer review history

# Peer review: Referee response for version 1

**DOI:** 10.5256/f1000research.12998.r29182

**Published:** 2018-01-19

**Authors:** Antonio C. Scarano

**Affiliations:** 1 Department of Medical, Oral and Biotechnological Sciences, University of Chieti-Pescara, Chieti, Italy

This manuscript reports an interesting study evaluating Er:YAG compared with conventional dental drilling by micro-computed tomographic and histological techniques in Ovine femoral heads model.

**Introduction**

 The introduction has been well written.

**Materials and Methods:**

 Specific Comments:

 Why did you choose to preparation of osteotomies in Ovine femoral heads model?

 How many samples were used? Did you do power analysis to see the required number of specimens per group?

**Discussion**

 The discussion has been well written.

I have read this submission. I believe that I have an appropriate level of expertise to confirm that it is of an acceptable scientific standard.

# Peer review: Referee response for version 1

**DOI:** 10.5256/f1000research.12998.r24950

**Published:** 2017-08-22

**Authors:** Robert A. Convissar

**Affiliations:** 1 New York Hospital Queens, New York, NY, USA

Excellent paper. Well written. Well researched. Excellent methodology and scientific protocol. The authors should be proud of their work.

 I would like to see a comparison of this to ErCrYSGG as a next step. My only comment is the age of the lambs-was the age appropriate and comparative to a human adult? If the age of the lamb was young-comparative to a child or teen, the results might be quite different with a more mature specimen.

I have read this submission. I believe that I have an appropriate level of expertise to confirm that it is of an acceptable scientific standard.
